# Preliminary evidence for altered motion tracking-based hyperactivity in ADHD siblings

**DOI:** 10.1186/1744-9081-10-7

**Published:** 2014-03-13

**Authors:** Verena Reh, Martin Schmidt, Winfried Rief, Hanna Christiansen

**Affiliations:** 1 Department of Clinical Psychology, University of Marburg, Marburg, Germany; 2 Department of Clinical Child and Adolescent Psychology, University of Marburg, Marburg, Germany

**Keywords:** Attention-Deficit/hyperactivity disorder, ADHD, Intermediate phenotype, Hyperactivity, Quantified behavior test, QbTest

## Abstract

**Background:**

It is well-established that ADHD children have deficits in executive functions such as performance variability and sustained attention. It has been suggested that these deficits are intermediate phenotypes. Hyperactivity, a core symptom of ADHD, has not yet been explored as a potential intermediate phenotype in ADHD. The computerized Quantified behavior Test (QbTest) is a combined continuous performance and activity test that assesses hyperactivity, inattention, and impulsivity separately. The aim of the present study was to (1) investigate the utility of objectively measured motor activity as a potential intermediate phenotype in ADHD, and (2) explore intermediate phenotypes for ADHD at the factor instead of single variable level.

**Method:**

Forty-five ADHD children, 22 non-affected siblings, and 45 unrelated controls with no family history of ADHD performed the QbTest. Effects of familiality as well as influences of age and gender on QbTest symptom dimensions were tested.

**Results:**

ADHD children showed the greatest impairments on all three QbTest factors, followed by their non-affected siblings, with control children showing the lowest scores. Group differences between the non-affected siblings and controls were only significant for the motion tracking-based Hyperactivity factor. Results were independent of age and gender.

**Conclusion:**

Hyperactivity assessed by a motion tracking system may be a useful intermediate phenotype in ADHD. Prospective research should use larger samples to further examine the QbTest factors, especially the motion tracking-based Hyperactivity factor which may be a candidate for an intermediate phenotype in ADHD.

## Introduction

Attention-Deficit/Hyperactivity Disorder (ADHD) is characterized by a triad of symptoms— age-inappropriate levels of hyperactive, inattentive, and impulsive behavior—that lead to severe impairments [1]. ADHD is present in up to 5% of school-aged children independent of cultural background [2-4]. It is well-established that there is a strong genetic basis for the disorder, and heritability estimates suggest that over 70% of the phenotypic variability in ADHD is due to genetic factors [5]. However, the complex etiological pathways from polygenetic origin to gene-environment interactions to the heterogeneous phenotype of ADHD are still not well understood [6].

Intermediate phenotypes have proven especially helpful for unraveling complex etiologies of psychiatric disorders [7]. Intermediate phenotypes are quantifiable heritable constructs that express an individual’s likelihood of developing or displaying symptoms of a given disease [8,9]. They can be studied at the neurophysiological, neuroanatomical, and/or neuropsychological level. Intermediate phenotypes are thought to be situated somewhere on the continuum of the genes underlying the disorder to diagnostic categories describing the manifest symptoms (phenotype). Identifying intermediate phenotypes can reduce heterogeneity at the symptom level and could help clarify classification and diagnosis of ADHD. Moreover, intermediate phenotypes may help characterize early predictors of ADHD that could lead to more refined treatments and primary prevention strategies [10,11]. Finally, intermediate phenotypes provide quantifiable measures that can advance genetic analysis of the disorder [7,12].

Several key criteria have been proposed for intermediate phenotypes (i.e., endophenotypes) in ADHD, including (1) a robust association with the disorder, (2) evidence of heritability, and (3) unaffected family members of affected individuals showing a higher rate of intermediate phenotype expression than individuals from the general population [8,10]. Additionally, the importance of quantitative instead of dichotomized intermediate phenotype measures as well as reliability and stability over time have been emphasized [10].

To date, research on neuropsychological intermediate phenotypes for ADHD has primarily focused on executive dysfunctions (ED) such as sustained attention, response control, and performance variability. There is considerable evidence for ED, like reduced inhibitory control and sustained attention, in ADHD children [13]. In particular, reaction-time (RT) variability and accuracy parameters like omission and commission errors have been identified as useful neuropsychological intermediate phenotypes for ADHD [10,14,15]. Accordingly, Go/No-Go tasks, commonly used to assess ED [16], but also actigraph measures, which are direct recordings of an individual’s body movements [17], are useful measurement techniques for exploring intermediate phenotypes for ADHD, although motion tracking-based hyperactivity has not yet been investigated as a potential intermediate phenotype.

Increased motor activity is a core feature of ADHD and predicts (beyond the age of four) a diagnosis of ADHD at age nine [18]. Objectively measured activity in ADHD has a decisive prognostic value [11], shows significant heritability estimates [17], and is an important source of information for studies that aim to improve ADHD phenotype definitions [19]. However, current models of ADHD often neglect the role of hyperactivity. This has been criticized because hyperactive symptoms are clinically most relevant as they are associated with a wide range of severe negative outcomes [1,20]. Moreover, hyperactivity seems to be the only empirically documented symptom that uniquely distinguishes children diagnosed with ADHD from those diagnosed with other childhood disorders [21].

The QbTest [22] is a combined Go/No-Go and activity test that objectively assesses the three core symptoms of ADHD. Single parameters such as RT variability, one of the identified intermediate phenotypes (see above), are available in addition to factor scores that correspond with the core symptoms [23]. This fulfills one important recommendation for how to refine research on ADHD intermediate phenotypes, because the use of factor scores increases statistical power by aggregating data and reducing error variance [24].

Using factor scores to aggregate data has been shown to be a highly effective strategy in neurogenetic research [25] and clinical research on familial neuropsychological deficits in schizophrenia [26].

The present study investigates the following three technically assessed QbTest factor scores as potential markers to improve phenotype definition: Inattention, Hyperactivity, and Impulsivity [23]. To our knowledge, this is the first study to investigate whether objectively measured hyperactivity constitutes a suitable marker for ADHD. Previous studies concerning objectively measured activity levels have suggested a genetic basis for hyperactive behavior [17] and showed that objectively measured hyperactivity variables are stable over time regardless of diagnostic status [11], making them ideal for research addressing intermediate phenotypes. Moreover, research has identified single variables like RT variability and omission and commission errors as useful neuropsychological intermediate phenotypes in ADHD [14,15,24], although these variables have not yet been studied at a factor level. To determine whether QbTest factors are suitable risk markers for future studies, we compared QbTest results from ADHD children, their non-affected siblings, and a healthy matched control group. We expect that (1) children with ADHD will significantly differ from control children across all three QbTest factors (i.e., ADHD children will show greater impairments than controls), indicating an association between QbTest factor scores and ADHD, (2) a similar pattern will be observed for non-affected siblings, who should show intermediate impairments (i.e., between ADHD and healthy control children), and (3) non-affected siblings will have significantly greater impairments than controls. Finally, we tested whether group differences are independent of age and gender.

## Method

### Sample

All participants were recruited in the context of a therapy study on ADHD at the department of clinical psychology and psychotherapy, Philipps University, Marburg, Germany for details on this study, see [27]. Families with a child suspected of having ADHD or parents of children who were already pre-diagnosed by a local pediatrician registered their child at the outpatient clinic of the department. Healthy control children were recruited from a local elementary school. Control children were required to have no formal or suspected ADHD diagnosis and no family history of ADHD. All children had to be 7–16 years old at the beginning of the study. Exclusion criteria included autism, IQ below 80, brain disorders, and any genetic disorder that mimics ADHD. Ethical approval for this study was obtained from the Department of Psychology, Philipps University Marburg, Germany. All parents and children gave written and verbal informed consent.

In total, 112 children (60.7% male) aged 7–16 years (mean age = 9.8 years, SD = 2.1 years) were included in the study. The sample consisted of 45 ADHD children, 22 non-affected siblings, and 45 healthy controls (see Table 1). A power analysis (G-Power) revealed that with the present sample sizes, and an alpha of .05, differences between control children and siblings with medium to large effect sizes (d > .65) could be detected with .8 power (differences between control and ADHD children were assumed to be larger and hence were not considered in the power analyses). Medium to large effect sizes for comparisons between ADHD siblings and healthy controls have been reported (Slaat-Willemsen et al. [13]). For small to medium effect sizes, the alpha-threshold would have to be raised to .14 to achieve satisfactory power of .8.

**Table 1:** Sample characteristics

	**Children with ADHD (n = 45)**		**Non-affected siblings (n = 22)**		**Controls (n = 45)**		**ANOVA**	
	**M**	**SD**	**M**	**SD**	**M**	**SD**	**F**_**2**_**,109**	**p**
Age in years	9.2	1.7	11.2	3.2	8.9	1.2	50.3^a^	< .001
% male	77.8		59.1		44.4		10.5^a^	.005
Conners parents DSM-IV								
Inattentive	64.1	11.8	57.1	13.4	47.4	7.6	26.8	<.001^b^
Hyperactive-impulsive	70.0	9.5	51.0	13.6	52.7	8.8	38.8	<.001
Total	70.0	7.3	55.6	11.3	51.3	7.0	61.8	<.001^c^
Conners’ teacher DSM-IV								
Inattentive	63.2	6.1	55.4”	10.0	52.9^e^	7.7	16.4	<.001^c^
Hyperactive-impulsive	61.5	8.6	50.1^d^	3.5	53.5^e^	6.9	13.2	<.001^c^
Total	64.3	7.3	55.4^d^	8.8	52.4^e^	7.2	20.8	<.001^c^

*Note.* ADHD = Attention-deficit/hyperactivity disorder; DSM-IV = *Diagnostic and Statistical Manual for Mental Disorders* (4th edition).

^a^χ *2*.

^b^The ADHD and the non-affected siblings group differ significantly from the healthy control group (*p* < .05).

^c^The ADHD group differs significantly from the non-affected siblings group and from the healthy control group (*p* < .05).

^d^Mean and SD of Conners’ teacher ratings only available for 10 of the non-affected siblings.

^e^Mean and SD of Conners’ teacher ratings only available for 26 of the healthy controls.

In the ADHD group, 91% of the children had a diagnosis of the combined subtype according to the *Diagnostic and Statistical Manual of Mental Disorders* DSM-IV, American Psychiatric Association; [28], 9% fulfilled the criteria for the predominantly inattentive subtype, and none of the children had the predominantly hyperactive/impulsive subtype. Comorbidity rates in the ADHD group were high, with 46% of children having at least one comorbid disorder. Twenty-nine percent were diagnosed with Oppositional Defiant Disorder (ODD), 9% with Enuresis Nocturna, 7% with anxiety disorders, and 3% with tic disorders.

The ADHD group significantly differed from the non-affected sibling and healthy control groups with respect to gender (*χ*^*2*^_*(2)*_ = 10.5, *p* = .005). In the ADHD group, 78% of the children were male, while only 59% and 44% were male in the non-affected sibling and control groups, respectively. In addition, the non-affected sibling group differed from the other two groups in age (*χ*^*2*^_*(2)*_ = 50.3, *p* < .01), as siblings tended to be older than the ADHD and control children. We therefore used age- and gender-adjusted QbTest values (Q-values) to calculate the QbTest factor scores, and also included both variables as covariates.

### Procedure and materials

ADHD children and their sibling were assessed by experienced and well-trained staff at the department of psychology. Children were motivated with small breaks between tasks and a game at the end of the testing session. Children who were diagnosed with ADHD were offered to participate in a current therapy study [27]. Assessment of control children was performed in a similar way. All control children received a cinema gift coupon (10 € value) for participation.

Parents and teachers of all children were asked to fill out the German version of the Conners’ 3rd rating scales [29]. Parent and teacher ratings (T-scores) greater than 63 on the total symptom subscale are regarded as clinically relevant. A biographical parent questionnaire covering demographic characteristics, family history of psychological or psychiatric disorders, and information on child development was completed for all children. Furthermore, a semi-structured, standardized clinical interview Kiddie-SADS; German version; [30] was conducted with all ADHD children. The Kiddie-SADS covers all psychological/psychiatric disorders according to the DSM-IV, and if symptom areas were screened as positive, the long version of the interview was performed. The Kiddie-SADS was not administered to non-affected siblings or controls, who were regarded as non-affected if they scored lower than 63 on either the Conners’ parent or teacher rating scale and if no history of behavior problems was reported in the biographical parent questionnaire.

The neuropsychological QbTest was administered to all participating children. Children in the ADHD group that were on stimulant medication were required to be off medication at least 48 hours prior to testing. The QbTest is a combined continuous performance (CPT) and activity test [22] that assesses the three core symptoms of ADHD. In general, the three QbTest factors—Hyperactivity, Inattention, and Impulsivity—show adequate construct and discriminant validity [23]. Qbtech© provides two different test versions of the test, one for children aged 6–12 years, and an adolescent/adult version 12-60 years. For both test versions, separate norms for male and female participants, and age-groups (per year in the children’s version, per decade in the adult version) are available. According to the test manual [22], the children’s version is based on normative data from 576 children (262 male, 314 female). For the adult version, normative data is based on data from 731 adults (360 male, 371 female).

### Statistical analysis

All analyses were conducted using SPSS 19.0. All variables were examined for accuracy of data entry and missing values prior to the analyses. No missing values were found. The Kolmogorov-Smirnov test did not reach significance for any of the three factors used as dependent variables in the multivariate analyses, thus normal distribution of the data can be assumed.

To control classification and separability of the three groups, we conducted t-tests for each group to determine whether group means were significantly different from our cut-off score in the Conners’ Parent questionnaire (T = 63; percentile = 90.3).

The three QbTest factors were computed according to factor analytic results [23]. Age- and gender-normed Q-values (similar to z-values) were used to calculate factor scores (see Table 2). To test group differences, a multivariate ANOVA (MANOVA) was performed with the QbTest factor scores as dependent variables and group (ADHD, non-affected sibling, healthy control) as a between subjects factor. Trends are reported as *p* < .08 and significance as *p* < .05. Pair-wise comparisons were conducted to further explore group differences and confirm the locus of effects. Effect sizes (η_p_^2^) are reported when appropriate and interpreted according to Cohen [31]; small: 0.01 ≥ η^2^; medium: 0.06 ≥ η^2^; and large: η^2^ ≥ 0.14 effects. For pair-wise comparisons, we used the post-hoc Scheffé test because it is most strict when sample sizes vary across groups. Additionally, non-parametric statistics (overall Kruskal-Wallis-Test for independent samples and post-hoc Mann-Whitney U-tests) were performed to test intergroup differences and thereby control multivariate test results. For non-parametric tests, *r* is reported as the effect size and is interpreted as 0.1 = small, 0.3 = medium, and 0.5 = large [32].

**Table 2:** Group differences in QbTest factor scores and QbTest single variables (T-values)

**Measure**	**ADHD (n = 45)**	**Siblings (n = 22)**	**Controls (n = 45)**	**MANOVA**		
	**Mean (SD)**	**Mean (SD)**	**Mean (SD)**	**F**_**2,109**_	**Sig.**	**η**_**p**_^**2**^
**QbTest factors**						
(1) Hyperactivity	53.87 (8.5)	51.55 (10.4)	45.28 (9.6)	9.94	<.00l^**^	.154
(2) Inattention	55.78 (8.7)	47.0 (9.5)	46.33 (8.2)	15.47	<.001^**^	.221
(3) Inipulsivity	54.93 (10.5)	49.57 (10.5)	44.86 (5.6)	14.57	<.001^**^	.211
**QbTest variables**						
Time active (1)	52.91 (7.6)	50.94 (10.3)	46.56 (10.9)	5.07	.008^**^	.084
Distance (1)	53.24 (9.5)	52.38 (11.0)	45.49 (10.9)	8.95	<.001^**^	.135
Area (1)	54.03 (8.0)	52.09 (10.5)	44.81 (9.5)	12.17	<.001^**^	.182
Microevents (1)	53.78 (9.7)	51.16 (10.3)	46.07 (9.0)	6.66	.002^**^	.108
Motion Sin1icitv (1)	54.05 (8.4)	50.32 (10.7)	45.74 (9.7)	8.59	<.001^**^	.139
Omission errors (2)	54.91 (8.2)	51.83 (11.6)	44.51 (7.8)	15.56	<.001^**^	.228
Reaction time (2)	53.69 (8.8)	44.08 (10.0)	50.07 (7.9)	11.0	.001^**^	.142
Reaction time variation (2)	55.65 (8.2)	47.31 (9.90)	45.96 (9.2)	15.0	<001^**^	.211
Commission errors (3)	53.52 (9.0)	51.01 (11.5)	45.54 (8.2)	8.7	.001^**^	.136
Multiresponse (3)	56.04 (10.9)	48.24 (9.2)	44.53 (4.7)	20.27	<.001^**^	.277
Anticipatory (3)	52.5 (12.3)	49.75 (9.7)	47.32 (6.2)	3.15	.046^*^	.055

*Note.*$\eta_{p}^{2}$ = partial Eta squared.

*p = .05.

**p = .001.

Even though effects of age and gender should have minimal influences because age- and gender-normed Q-values were used to calculate factor scores, we still performed a MANCOVA with the QbTest factor scores as dependent variables, group (ADHD, non-affected sibling, healthy control) as a between subject factor, and age and gender as covariates.

Finally, to further explore effects of familiality, we conducted trend analyses across the three groups to test whether non-affected siblings show intermediate impairments between ADHD children and healthy controls. A linear trend in the absence of a residual quadratic trend would indicate familiality of the given construct. A residual quadratic trend would indicate that the non-affected siblings were more similar to either the ADHD or control group [15].

## Results

### Group differences in Hyperactivity, Impulsivity, and Inattention QbTest factor scores

The ADHD group scored significantly higher than 63 on the Conners’ Parent ADHD index (Mean = 70.0; SD = 7.3; *p* < .001). The sibling group (Mean = 56.2; SD = 11.3; *p* = .014), and control group (Mean = 51.3; SD = 7.0; *p* < .001) both scored significantly lower than 63.

The MANOVA performed on the QbTest data showed a large and significant main effect of group (F_(2,109)_ = 7.75, *p* < .01, $\eta_{p}^{2}$ = .177), indicating that groups differed in mean hyperactivity, inattention, and behavioral impulsivity scores. There were significant differences between groups for all three factors, and the effect sizes were large (Inattention: F_(2,109)_ = 15.47, *p* < .01, $\eta_{p}^{2}$ = .221; Impulsivity: F_(2,109)_ = 14.57, *p* < .01, $\eta_{p}^{2}$ = .211; Hyperactivity: F_(2,109)_ = 9.94, *p* < .01, $\eta_{p}^{2}$ = .154; see Table 2). As predicted, ADHD children had the highest scores (greatest impairment) for all three symptom dimensions, followed by their non-affected siblings, and control children had the lowest scores. These results were confirmed by non-parametric analyses (Inattention: χ^2^_(2)_ = 24.4, *p* < .01; Impulsivity: χ^2^_(2)_ = 22.7, *p* < .01; Hyperactivity: χ^2^_(2)_ = 16.5, *p* < .01).

**Figure 1:**
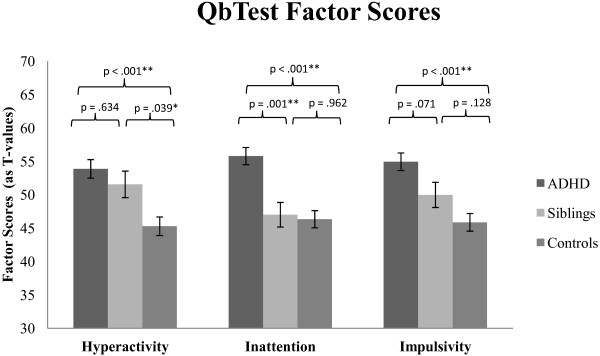
MANOVA results for the Hyperactivity, Inattention and Impulsivity Factor (bars depict group means and standard errors of the mean).

Pair-wise comparisons (post-hoc Scheffé tests) confirmed significant group differences between the ADHD and control group for all three factors (*p*s < .01). This was supported by results from the non-parametric Mann-Whitney-U test and effect sizes ranged from medium to large (Hyperactivity: U = 521.00, *p* < .01, *r* = −0.42; Inattention: U = 433.00, *p* < .01, *r* = −0.49; Impulsivity: U = 426.00, *p* < .01, *r* = −0.5). As expected, ADHD children were significantly more hyperactive than control children while performing the QbTest, and also showed more impulsive and inattentive behaviors (see Figure 1).

Hyperactivity factor scores were significantly different between non-affected siblings and controls (*p* = .039), such that non-affected siblings showed an intermediate level of motor activity between their ADHD siblings and healthy controls. This effect was confirmed by non-parametric results that had a medium effect size (U = 313.5, *p* = .015, *r* = −0.3), and can thus be interpreted as preliminary evidence for the utility of the Hyperactivity factor as a candidate intermediate phenotype for ADHD. The difference between the non-affected sibling and healthy control groups did not reach significance for the Impulsivity factor (*p* = .128). The non-parametric Mann-Whitney-U test revealed a trend towards a significant difference in behavioral impulsivity between non-affected siblings and healthy controls (U = 360.0, *p* = .071, *r* = −0.22). Furthermore, a post-hoc Scheffé test revealed no significant difference between the non-affected sibling and control groups on Inattention factor scores (*p* = .962), and group means for those two groups were very close, as shown in Figure 1. There were significant group differences between ADHD children and their non-affected siblings (*p* < .01), indicating that non-affected siblings were more similar to the healthy controls than to their biological ADHD siblings in terms of inattention.

### Effects of age and gender

The MANCOVA revealed the following results for age and gender. Hyperactivity factor scores were not significantly influenced by either age (F_(1,107)_ = 0.61, *p* = .435, $\eta_{p}^{2}$ = .006) or gender (F_(1,107)_ = 1.66, *p* = .201, $\eta_{p}^{2}$ = .015), nor were Inattention (age: F_(1,107)_ = 1.79, *p* = .184, $\eta_{p}^{2}$ = .016; gender: F_(1,107)_ = 1.46, *p* = .230, $\eta_{p}^{2}$ = .013) or Impulsivity factor scores (age: F_(1,107)_ = 0.48, *p* = .226, $\eta_{p}^{2}$ = .014; gender: F_(1,107)_ = 0.43, *p* = .514, $\eta_{p}^{2}$ = .004).

### Trend analyses

Trend analyses for Hyperactivity factor scores revealed a strong linear trend (F_(2,109)_ = 19.09, *p* < .01) without a quadratic trend (F_(2,109)_ = 0.79, *p =* .38) indicating that, as predicted, the non-affected sibling group showed an intermediate level of motor activity compared to the more hyperactive ADHD group and the less hyperactive healthy control group. Hence, motor activity showed evidence of familiality. For Inattention factor scores, there was a linear trend (F_(2,109)_ = 26.95, *p < .01*), and the quadratic trend also reached significance (F_(2,109)_ = 3.98, *p =* .05). Inattention levels were very similar between the non-affected sibling and control groups (see Table 2), and thus there was no evidence of familiality for inattention. Finally, there was a linear trend for Impulsivity factor scores (F_(2,109)_ = 29.11, *p < .*01) in the absence of a quadratic trend (F_(2,109)_ = 0.02, *p < .*88), indicating that the three groups showed the typical intermediate phenotype pattern across behavioral impulsivity levels.

## Discussion

The present study was a pilot study designed to investigate the utility of QbTest factor scores (i.e., technically assessed Hyperactivity, Inattention, and Impulsivity) as potential intermediate phenotype markers for ADHD. This is the first study to explore motion tracking-based motor activity in ADHD siblings. Moreover, we examined neuropsychological intermediate phenotypes for ADHD at the factor, rather than single variable, level for the first time. We hypothesized that ADHD children would show the greatest impairments in task performance and that their non-affected siblings would show intermediate impairments compared to a control group of children with no family history of ADHD.

Confirming our first hypothesis, children with ADHD showed substantially greater impairments compared to children from the control group across all three QbTest factors. They were more inattentive and impulsive, and showed higher levels of motor activity while performing the QbTest.

The Hyperactivity factor consisted of the following five QbTest variables: Time Active, Distance, Area, Microevents, and Motion Simplicity [22,23]. ADHD children had significantly higher motor activity scores on all five QbTest variables, thus showing higher frequency of movement (indexed by a high percentage of Time Active) and larger body movement amplitudes (indicated by Distance, Area, Microevents, and Motion Simplicity scores) than controls. These findings are consistent with previous studies that used motion tracking-based measurement techniques and reported that ADHD children are more active than healthy control children during a CPT [11,19].

The Inattention factor was composed of three QbTest variables: RT, RT variability, and Omission errors. ADHD children reacted more slowly and variably and they also committed more omission errors than did children in the control group. RT variability and omission errors have repeatedly been shown to be higher in children with ADHD compared to healthy controls [14,15,24]. Thus, our results are in line with the majority of studies comparing ADHD children and controls with respect to attention variables.

The Impulsivity factor consisted of three QbTest variables: Commission errors, Multiresponses, and Anticipatory responses. ADHD children committed more commission errors (false alarms), and had a higher percentage of multiresponse and anticipatory responses than controls. Commission errors have been defined as a measure of deficient response inhibition and have been shown to be substantially elevated in ADHD children compared to controls [13]. This is consistent with our Impulsivity factor score results.

We also hypothesized that children from the non-affected sibling group would show intermediate impairments—between ADHD and healthy control children—but would be significantly different from controls.

We found a strong linear trend across group means for the motion tracking-based Hyperactivity factor. As expected, non-affected siblings showed an intermediate level of motor activity between ADHD children (highest activity scores) and controls (lowest activity scores). Moreover, there was a significant difference between the non-affected sibling and control groups. Taken together, the presence of increased motor activity not only in ADHD children but also in their non-affected siblings indicates that the QbTest Hyperactivity factor fulfills two important criteria for an intermediate phenotype measure [24]: it co-occurred with the disorder and it was manifest in individuals who carry genes for ADHD but do not express the disorder itself.

We found a linear trend across groups, with ADHD children showing greatest, non-affected siblings showing intermediate, and control children showing the least impairment for the Inattention and Impulsivity factors. Group means for the non-affected sibling and control groups did not significantly differ for either the Inattention or Impulsivity factors. Note, however, that the present study may have been underpowered for detecting small to medium effects between non-affected siblings and control children (see power analyses in the method section), and hence subsequent studies should investigate whether QbTest factors are intermediate phenotypes using larger groups.

Results also showed that non-affected siblings performed very similarly to children from the control group with respect to the Inattention factor, which was supported by a significant quadratic trend. This contrasts with other findings suggesting that RT variability [14], omission errors [15], and commission errors [13] are in fact candidates for intermediate phenotypes in ADHD. However, these studies emphasized the relevance of motivational factors in ADHD, especially for RT variability. The influence of motivational factors on performance variation is beyond the scope of this paper, but further studies should assess motivational aspects because they might explain the discrepancy between our results and previous studies.

Furthermore, we explored whether group differences were independent of age and gender. Results clearly showed that none of the three QbTest factors were influenced by age or gender, indicating that adjusting by age and gender norms provided in the QbTest was successful. Finally, it should be noted that while intermediate phenotypes in ADHD can be assessed and established on different levels (neurophysiological, neuroanatomical, and neuropsychological levels), these lie on a continuum from the genetic underpinnings of the disorder to biologically rooted intermediate phenotypes to neuropsychological variables and factor scores to the observation of behavior representing the full heterogeneity of the ADHD phenotype. The QbTest factor scores are based on the neuropsychological level of the disorder and may represent a marker for ADHD that could ultimately help to improve phenotype definition.

### Limitations

The following limitations of this study should be noted. First, as discussed above, age and gender distributions differed between the groups in our study. Boys were overrepresented in the ADHD group, and mean age was higher in the non-affected sibling than ADHD and control groups. However, analyses controlling for age and gender did not reveal significant influences of age and gender, and the higher number of boys in the ADHD group reflects the male to female ratio in ADHD [2-4].

Second, we did not administer the clinical interview to non-affected siblings and controls. Additionally, Conners’ Teacher Questionnaires were missing for some children in the non-affected sibling and control groups. However, known formal diagnosis of ADHD and other childhood disorders were assessed in the biographical parent questionnaire and participation in the control group was explicitly advertised as seeking children without any ADHD-related behavior problems. Nevertheless, five children in the non-affected sibling group and two children in the control group showed high Conners’ Parent ratings (T > 63). As parent ratings of ADHD behavior and QbTest factor scores were significantly correlated (Pearson’s Correlation: .24 < *r* > .44), it is likely that non-affected siblings and controls who show elevated QbTest scores also have higher ADHD behavior ratings than children with lower QbTest scores. As described above, we controlled classification and separability of the three groups by testing group means against our Conners’ Parent questionnaire cut-off score (T = 63). The ADHD group scored significantly higher, while the other two groups scored significantly lower, than the cut-off score. Thus, overall, the groups adequately differed with respect to parent ratings of ADHD-related behavior.

Finally, due to relatively small groups, the reported results are preliminary and need to be confirmed in larger samples. Future studies should further explore (1) the utility of technically assessed motor activity as an intermediate phenotype in ADHD, and (2) the advantage of neuropsychological factor scores over single variable scores.

## Conclusions

Establishing risk markers in ADHD is highly desirable as it could reduce heterogeneity at the symptom level and thereby help clarify ADHD classification and diagnosis. The present study is the first to explore the utility of factor scores from the neuropsychological QbTest as potential intermediate phenotypes for ADHD. ADHD children exhibited the greatest impairment on all three factors, followed by their non-affected siblings, with control children showing the least impairment. However non-affected siblings only differed significantly from controls on the motion tracking-based Hyperactivity factor. Results were independent of age and gender. This provides important preliminary information concerning the utility of motor activity as a new intermediate phenotype for ADHD.

## Competing interests

The authors declare no competing interests.

## Authors’ contribution

VR planned the study, recruited participants, analysed the data and wrote a first draft of the MS. MS planned the study, recruited participants, and proof-read the MS. WR supervised study planning and proof-read the MS. HC supervised the study, worked on the MS draft and the final MS version, and proof-read the MS. All authors read and approved the final manuscript.
